# Reexamining Sexual and Species Recognition in Butterflies: The Partner‐Hazard Space Framework

**DOI:** 10.1002/ece3.74101

**Published:** 2026-08-07

**Authors:** Tsuyoshi Takeuchi, Daisuke Muramatsu

**Affiliations:** ^1^ Entomological Laboratory, Graduate School of Agriculture Osaka Metropolitan University Sakai Osaka Japan; ^2^ Research Institute for Humanity and Nature Kyoto Kyoto Japan

**Keywords:** mate choice, same‐sex sexual behavior, sexual recognition, sexual selection, species recognition

## Abstract

Generally, intra‐ and inter‐sexual interactions have been considered qualitatively different processes. However, same‐sex sexual behavior is widespread in the animal kingdom, challenging this assumption. Recently, a hypothesis was proposed that regards territorial contests of butterflies, where two males chase each other without apparent physical attack, as an initial phase of mating behavior. Building on this idea, we reviewed research on sexual and species recognition in butterflies to assess the extent to which they recognize the same sex and species. We found no evidence that butterflies recognize the same sex, and consequently, no evidence that they recognize their own species (the set of the same sex and the opposite sex). Phenomena previously interpreted as sexual or species recognition were indistinguishable from those interpreted as mate choice. In addition, although butterflies tend to escape from looming objects, there is no evidence that they recognize predators at the species level. Thus, known butterfly interactions can be explained by assuming that they possess a sexual‐partner template (that elicits mating behavior) and a hazard template (that elicits escape responses). Within this framework, other animals can be located in a space according to their similarity to sexual‐partner and hazard templates. We term this framework the Partner‐Hazard Space (PHS). Under PHS, intra‐ and most interspecific interactions of butterflies can be understood as various phases of sexual behavior or escape responses to hazards. PHS provides a parsimonious hypothesis for interpreting butterfly interactions without assuming recognition of sexual rivals or species. Furthermore, PHS also provides a simple explanation for the evolution of conspicuous sexual and species‐specific traits in butterflies.

## Graphical abstract

Our review found no evidence that butterflies recognize the same sex or their own species. Behaviors previously interpreted as sexual or species recognition are indistinguishable from those interpreted as mate choice. Butterfly interactions can therefore be explained by assuming a sexual‐partner template that elicits mating behavior and a hazard template that elicits escape responses.

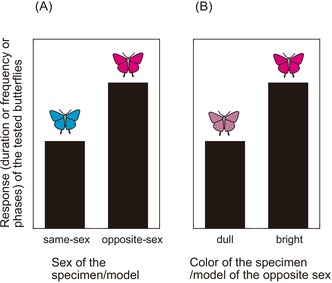

## 1 Introduction

Sexual traits and behaviors have attracted the interest of researchers, leading to the development of many sexual selection theories (Kuijper et al. 2012; Parker 2014). Sexual selection involves intra‐ and inter‐sexual selection (Jones and Ratterman 2009). Research on intra‐sexual selection has primarily focused on mate competition. Contest models based on game theory have developed since the publication of the seminal paper on animal conflict by Maynard Smith and Price (1973). In contrast, research on inter‐sexual selection has focused mainly on mate choice. Runaway coevolution and the good genes hypothesis are among the most well‐known theories explaining mate choice (Fisher 1930; Zahavi 1975; Grafen 1990). Over the past quarter century, sexual conflict, defined as differences in evolutionary interests between males and females, has also become a major focus of study (Chapman et al. 2003).

Most sexual selection theories emphasize intra‐sexual competition, inter‐sexual mate choice, and sexual conflict. In doing so, these theories have often overlooked the process by which individuals choose their sexual partners from among conspecifics of both sexes and heterospecifics. Yet, animals sometimes court and copulate with heterospecifics resulting in reproductive interference (Gröning and Hochkirch 2008; Shuker and Burdfield‐Steel 2017). Moreover, males sometimes exhibit sexual behavior toward conspecific males (Bailey and Zuk 2009; Caballero‐Mendieta and Cordero 2012; Scharf and Martin 2013). These findings have often been attributed to uncertainty in sexual or species recognition. Such cognitive limitations are often ignored in sexual selection theories, despite their potential influence on the evolution of sexual traits and behaviors.

We think that the gap between sexual selection theories and empirical observations stems from overestimation of animals' cognitive abilities. Humans tend to assume that sexually reproducing animals clearly recognize the sexes of conspecifics. Indeed, animals must possess some form of opposite‐sex template to perform mating behavior, regardless of whether their recognition is accurate or inaccurate. In contrast, it is not evident that animals possess same‐sex templates, because mating behavior can occur without them. Consequently, it is also unclear whether animals possess templates of their own species (the set of the same and the opposite sexes).

According to Sherman et al. (1997), templates are internal representations of the characteristics of desirable or undesirable objects. Recognition occurs when phenotypes of objects match these templates closely enough. If animals possess templates of both sexes, what do the two sexes represent for them? Functionally, the opposite sex consists of potential sexual partners, whereas the same sex consists of potential sexual rivals. For clarity, we use this definition of both sexes. Since we cannot directly assess animal cognition, we can only infer it from animal behavior. Such a discussion may seem speculative; however, it has recently advanced our understanding of the behavior of certain animals, particularly butterflies.

Butterflies have been widely studied in the context of sexual selection due to their vibrant coloration and sexual dimorphism (Allen et al. 2011; Cannon 2020). However, certain aspects of their behavior remain enigmatic. In many butterfly species, males perch on leaves or the ground at a mating station to wait for passing females. When another male flies into the area, the perching male rushes toward it, and the two males chase each other without physical attack until one of them flees (Kemp and Wiklund 2001; Takeuchi 2017). Although these territorial contests have been analyzed using game theoretic models, they are difficult to explain from the perspective of individual selection because males appear to impose costs (energy or time) not on their opponent but on themselves. Takeuchi et al. (2016) proposed the erroneous courtship hypothesis (ECH), which interprets these territorial contests as an initial phase of courtship behavior directed toward the same sex due to their inaccurate sexual recognition. ECH reasonably explains why butterflies perform aerial displays that impose costs on themselves.

ECH is based on the specific assumption that territorial butterflies cannot discriminate the sexes of “conspecifics in flight” since it was constructed to explain territorial contests of butterflies. In this paper, we extend the ECH framework to all intra‐ and inter‐specific interactions of butterflies. We first review research on sexual and species recognition in butterflies and reexamine the extent to which butterflies recognize sexual rivals and species. Based on the results, we construct a cognitive model of butterfly behavior and evaluate the applicability of this framework.

## 2 Sexual Recognition in Butterflies

Studies on sexual recognition in butterflies have been conducted since the mid‐20th century (Stride 1956). The majority of these studies were originally designed to identify releasers of mating behavior and, in doing so, also provided information on sexual recognition. The identity of releasers themselves is not the focus of the present review. Instead, we ask whether butterflies recognize both sexes. Because sexually reproducing butterflies must possess some mechanisms for recognizing potential mates, we focus on experiments that allow assessment of sexual recognition, that is, whether butterflies recognize both sexes rather than on sensory cues underlying mate recognition.

Generally, the target butterflies were males, as they exhibit mating behavior much more actively than females, making it easier to design bioassays. Researchers have typically presented specimens (including living ones) or models of male and female butterflies to target butterflies and observed their responses (Table 1). In chemical studies, candidate materials are applied to specimens or models to test their effects. Researchers typically record the frequency of approaches and/or the duration of hovering over the specimens or models. If male courtship behavior consists of multiple phases, the most advanced phase displayed toward the specimens or models was documented.

**TABLE 1:** Studies examining sexual recognition in butterflies.

Species	Target sex	Presented objects	Recorded responses	Results	References
Papilionidae					
*Parnassius glacialis*	Male	Wings	Phases of mating behavior	+	Yamashita (1995)
*Graphium sarpedon*	Male	Dead specimens	Phases of mating behavior	+	Kato and Yoshioka (2003)
*Battus philenor*	Male	Dead specimens	Response duration	+	Rutowski and Rajyaguru (2013)
*Papilio xuthus*	Male	Wings	Phases of mating behavior	+	Yamashita (1995)
*Papilio machaon*	Male	Dead specimens	Phases of mating behavior	+	Takeuchi et al. (2019)
*Papilio demoleus*	Male	Wing dummies	Number of chases	+	Chen et al. (2023)
*Papilio protenor*	Male	Wings	Phases of mating behavior	+	Yamashita (1995)
*Papilio helenus*	Male	Wings	Phases of mating behavior	0	Yamashita (1995)
*Papilio polytes*	Male	Dead specimens	Phases of mating behavior	+	Ômura et al. (2020)
*Papilio polytes*	Male	Wing dummies with wing extract	Phases of mating behavior	+	Ômura et al. (2020)
*Papilio maackii*	Male	Wing dummies	Frequency of courtship	+	Chen et al. (2021)
Pieridae					
*Pieris rapae*	Male	Dead specimens	Number of approaches	+	Obara (1970)*
*Pieris rapae*	Male	Dead specimens	Number of interactions	+	Stoehr et al. (2016)
*Pieris protodice*	Male	Living specimens	Chase duration	+	Rutowski (1981)
*Pieris protodice*	Female	Living specimens	Chase duration	+	Rutowski (1981)
*Eurema lisa*	Male	Dead specimens	Number of contacts	+	Rutowski (1977b)
*Colias eurytheme*	Male	Wings attached to bodies	Number of approaches	+	Silberglied and Taylor (1978)
*Colias philodice*	Male	Wings attached to bodies	Number of approaches	+	Silberglied and Taylor (1978)
Lycaenidae					
*Neozephyrus japonicus*	Male	Wings	Staying time	+	Imafuku and Kitamura (2015)
*Chrysozephyrus smaragdinus*	Male	Wings	Staying time	+	Imafuku and Kitamura (2015)
*Polyommatus icarus*	Male	Dead specimens	Number of approaches	—	Lundgren (1977)*
*Plebejus argus*	Male	Dead specimens	Number of approaches	—	Lundgren (1977)*
*Pseudozizeeria maha*	Male	Dead specimens	Phases of mating behavior	0	Wago et al. (1976)*
*Pseudozizeeria maha*	Male	Living specimens	Phases of mating behavior	+	Wago (1978)*
*Pseudozizeeria maha*	Female	Wings	Duration of stay	+	Imafuku and Kitamura (2018)
*Tongeia fischeri*	Male	Dead specimens	Phases of mating behavior	0	Hisai et al. (2026)
Nymphalidae					
*Hypolimnas misippus*	Male	Wings attached to bodies	Phases of mating behavior	+	Stride (1956)*
*Cethosia cyane*	Male	Wings	Frequency of courtship	+	Li et al. (2017)
*Cethosia cyane*	Male	Living males treated with or without female‐specific compounds	Chase duration	+	Li et al. (2017)
*Morpho helenor*	Male	Wings	Frequency of interactions	0	Le Roy et al. (2021)
*Morpho achilles*	Male	Wings	Frequency of interactions	0	Le Roy et al. (2021)

*Note:* Results are summarized as follows: +, Tested butterflies responded more strongly to models of the opposite sex than to those of the same sex; −, they responded more strongly to models of the same sex than to those of the opposite sex; 0, they responded equally strongly to models of both sexes. Some studies published before 1980 did not include statistical analyses. For these studies, we simply compared the response strength to models of the same and opposite sexes. These studies are indicated by an asterisk.

A notable feature of butterflies is that they generally do not exhibit agonistic behaviors toward same‐sex specimens or models. This contrasts with other insects such as odonates, where males exhibit aggressive behaviors toward specimens of the same sex (Hooper et al. 2006; Schultz and Fincke 2009).

### 2.1 Males

**FIGURE 1:**
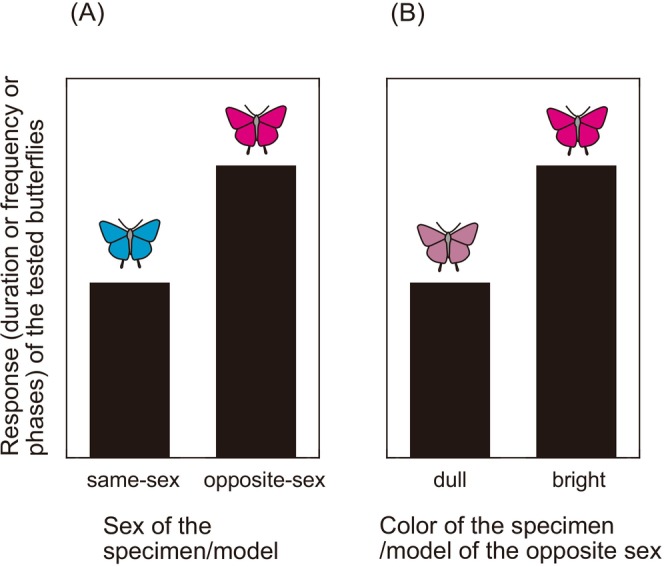
Typical results of experiments on sexual recognition (A) and mate choice (B). Although these results are often interpreted differently, the behavioral patterns are identical: Butterflies respond more strongly to one stimulus than to another in both cases.

We summarize our review in Table 1. Most studies consisted of multiple experiments and measured multiple behavioral variables. In such cases, we selected an experiment using models closest to real butterflies (living specimens>freshly killed ones>dried ones>wings only) and adopted behavioral variables directly assessing mating interest (phase of mating behavior>chase duration or the number of interactions). In most studies, males responded longer, more frequently, or showed more advanced courtship phases toward female specimens/models than toward male ones (Figure 1A) (Stride 1956; Obara 1970; Rutowski 1977b, 1981; Silberglied and Taylor 1978; Wago 1978; Yamashita 1995; Kato and Yoshioka 2003; Rutowski and Rajyaguru 2013; Imafuku and Kitamura 2015; Stoehr et al. 2016; Li et al. 2017; Takeuchi et al. 2019; Chen et al. 2021, 2023). Similarly, males generally responded more strongly to specimens/models with chemicals derived from females than to those with chemicals from males (Wago 1978; Li et al. 2017; Ômura et al. 2020). These results have often been interpreted as evidence of sexual recognition or sexual discrimination. In some cases, males responded equally to female and male specimens (Wago et al. 1976; Yamashita 1995; Le Roy et al. 2021; Hisai et al. 2026). Among these studies, we highlighted studies most suitable for investigating sexual recognition, where complete specimens of males and females are presented and the phases of mating behavior were recorded (Table 2). Generally, males are more likely to attempt copulation with female specimens than with male specimens.

**TABLE 2:** Studies most suitable for investigating sexual recognition, in which complete male and female specimens were presented and the phases of mating behavior were recorded.

Species	Target sex	Specimens	Copulation attempts toward female specimens	Copulation attempts toward male specimens	References
Papilionidae					
*Graphium sarpedon*	Male	Freshly killed	28/32	20/31	Kato and Yoshioka (2003)
*Papilio machaon*	Male	Freshly killed	1/10	1/10	Takeuchi et al. (2019)
*Papilio polytes*	Male	Freshly killed	46	5	Ômura et al. (2020)
Lycaenidae					
*Pseudozizeeria maha*	Male	Freshly killed	8	6	Wago et al. (1976)
*Pseudozizeeria maha*	Male	Live	24/24	4/20	Wago (1978)
*Tongeia fischeri*	Male	Dried	5	7	Hisai et al. (2026)

*Note:* Copulation attempts by tested butterflies toward female and male specimens are shown. Recording methods differed among studies. When the occurrence of copulation attempts was recorded for each trial, results are presented as fractions. When only the total number of copulation attempts was recorded, the raw counts are presented.

However, this interpretation is not the only possible explanation. Does a stronger response to the opposite sex necessarily indicate sexual recognition? In other words, is it necessary to assume that butterflies classify individuals as sexual partners and sexual rivals to explain these results? To explore this question, consider mate choice experiments. Suppose that in a butterfly species in which females exhibit variable traits (such as wing color), conspecific males are presented with specimens/models of a bright female and a dull female. If males respond more strongly to the bright female than to the dull female (Figure 1B), researchers typically interpret this as a preference for bright females (Wiernasz 1995; Burghardt et al. 2000; Ellers and Boggs 2003). In this case, the results are not evidence of sexual recognition, but rather indicate that males recognize both models as sexual partners and have a preference for one over the other.

This interpretation raises an important issue because Figure 1A and 1B are identical graphs; male responses are the same. Only the presented specimens/models are different. Just like Figure 1B, Figure 1A should be explained without assuming that males recognize sexual rivals: males respond to conspecific males as less attractive mating stimuli. This interpretation is favored by the principle of parsimony since it does not require the additional assumption that butterflies are able to recognize sexual rivals. Therefore, experimental results like those in Figure 1A (summarized in Table 1) are not evidence for sexual recognition but are evidence for mate preference.

It should be noted that even if the difference in responses is larger in Figure 1A than in Figure 1B, this conclusion does not change. Thus, quantitative meta‐analyses comparing the magnitude of response differences between sexual recognition experiments and mate choice experiments are not suitable for assessing the existence of sexual recognition. This consideration highlights a key issue: it may be misleading to interpret quantitative differences in behavior (intensity or frequency of courtship) as evidence of a qualitative concept (sexual recognition).

In a few butterfly species, males responded more strongly to male specimens than to female ones (Table 1; Lundgren 1977). We can explain such results as indicating that male colorful wings act as a supernormal stimulus of sexual partners to conspecific males. If butterflies were capable of recognizing the sexes of conspecifics, these results would be difficult to explain.

### 2.2 Females

Sexual recognition in female butterflies has rarely been examined because it is difficult to induce active mating behavior in females (e.g., see Wago 1977b). We found only two studies on the sexual recognition in female butterflies using the same methods applied to males (Table 1) (Rutowski 1981; Imafuku and Kitamura 2018). In both studies, females responded longer to male specimens/models than to female ones. Based on the same logic as in the previous section, these results indicate that females perceive conspecific females as less attractive partners. At present, we have found no study indicating that female butterflies are able to recognize sexual rivals.

## 3 Species Recognition in Butterflies

### 3.1 Recognition of Their Own Species

Since there is no evidence that butterflies recognize sexual rivals, there is likewise no basis for assuming that they recognize their own species (the set of sexual partners and rivals).

More fundamentally, the definition of species recognition remains obscure. It is highly unlikely that animals recognize each species individually as human biologists do. Standard textbooks of animal communication and behavioral ecology do not include a section on species recognition. Instead, they discuss forms of social recognition, including kin recognition, mate recognition, parent‐offspring recognition, and group recognition (Krebs and Davies 1997; Bradbury and Vehrencamp 2011).

Mendelson and Shaw (2012) argued that species recognition has been considered a stable part of evolutionary biology but is poorly defined and misleading. They pointed out the following. A common assumption in the literature on species recognition is that animals engage in a sequential process of mate choice, in which individuals must first choose the correct species, and then choose a high‐quality mate within that species. In this process, however, whether species indicators are processed before mate quality indicators is an intriguing hypothesis that has not been empirically tested. At present, species recognition is at most a process by which animals tend to choose conspecifics as mates. If one states that animals can distinguish between their own and other species, this statement should be rigorously tested. In this subsection, we examine species recognition in butterflies from this perspective.

**FIGURE 2:**
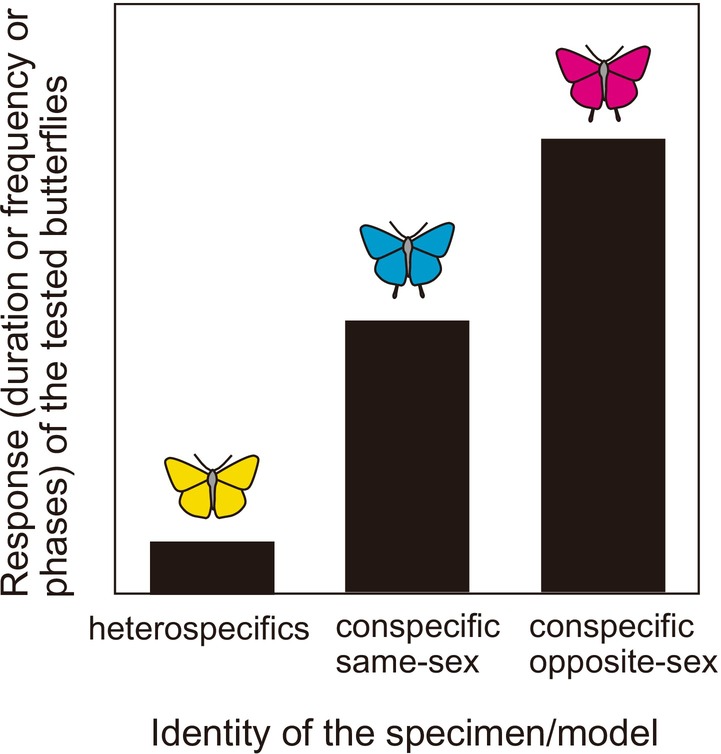
Typical results of experiments on species recognition. Butterflies often respond most strongly to conspecific individuals of the opposite sex, less strongly to conspecific individuals of the same sex, and least strongly to heterospecifics.

Like sexual recognition, species recognition has been studied in butterflies. Researchers have presented specimens or models (with candidate materials in chemical studies) of both conspecifics and heterospecifics (usually related species) to a target butterfly and observed male response. The frequency of approaches and/or the duration of hovering over each specimen or model was often recorded. In many cases, males responded for longer or more frequently to conspecific specimens/models than to heterospecific ones (Rutowski 1977b; Yamashita 1995; Jiggins et al. 2001; Fordyce et al. 2002; Ômura et al. 2020; Le Roy et al. 2021; Li et al. 2022; Klein and de Araújo 2023) (Figure 2). In some cases, males responded equally to conspecific and heterospecific specimens/models (Wago 1977a; Yamashita 1995), or even more strongly to heterospecific specimens/models (Le Roy et al. 2021; Klein and de Araújo 2023). The latter cases can be interpreted as responses to supernormal stimuli. As described above, these quantitative differences in responses can be explained as mate preference without invoking species recognition.

It is difficult to investigate species recognition in female butterflies as well as their sexual recognition. We found no studies demonstrating that female butterflies are able to recognize their own species. From the perspective of Mendelson and Shaw (2012), in which species recognition is viewed as a form of mate choice, there is some relevant evidence. Recent studies have shown that female butterflies prefer to mate with brighter conspecific males (Kemp 2007; Papke et al. 2007; Morehouse and Rutowski 2010; Rutowski and Rajyaguru 2013; Wedell and Kemp 2024) or males with intermediate‐sized eye spots on their wings (Robertson and Monteiro 2005). Several species rely on chemical cues for mate choice (Rutowski 1977a; Andersson et al. 2007; Nieberding et al. 2008; Honda et al. 2016, 2018; González‐Rojas et al. 2020; Gonzalez‐Karlsson and Grether 2021; Yoshimori et al. 2022). Ultimately, species recognition as a behavior distinct from mate choice has not been confirmed.

In summary, the behavioral evidence reviewed here can be explained without assuming that butterflies recognize their own species. What has previously been interpreted as species recognition is better understood as mate preference (Figure 2).

### 3.2 Predator Recognition

Few butterfly behaviors suggest that butterflies recognize heterospecific animals at the species level. Candidate behaviors include predator avoidance. Butterflies escape from certain animals, such as birds and humans, when attacked or approached (Chai and Srygley 1990; Pinheiro 1996; Kojima 2022). This raises the question of whether butterflies recognize predators at the species level.

Tinbergen (1972) reported that males of the grayling *Eumenis semele* exhibit fear responses to larger objects moving in front of them. Generally, approaching objects are perceived as hazardous by butterflies (Almbro and Kullberg 2008; Kojima 2022). However, to date, there is no evidence that butterflies recognize predators at the species level. This may be unsurprising, as escape behaviors are, by necessity, fast and robust; butterflies only need to escape from looming objects. It is well known that looming stimuli provoke escape behaviors in other insects and animals (Card 2012; Herberholz and Marquart 2012).

## 4 The Partner‐Hazard Space Framework

As reviewed above, sexual recognition and species recognition have often been loosely assumed rather than rigorously defined. To resolve this confusion, we propose a conceptual framework for butterfly cognition termed the “Partner‐Hazard Space” (PHS), in which other animals are represented according to their similarity to a sexual‐partner template and a hazard template. In the framework, templates are conceptually similar to releasers. We use templates because specific releasers are not identified in most butterfly species. Of course, identification of releasers is unnecessary to clarify what animals recognize. For example, performing mating behavior toward something is evidence of mate recognition. PHS is based on the following assumptions:

1. Butterflies possess a sexual‐partner template.
2. Butterflies do not recognize sexual rivals. Consequently, they do not recognize their own species (the set of potential sexual partners and sexual rivals).
3. Butterflies possess a hazard template.
4. Butterflies regard animals that are far from these two templates as, at most, obstacles

Assumption 1 is based on the fact that butterflies perform mating behaviors. Assumptions 2 and 3 are based on the conclusion of the previous sections. Assumption 4 follows from the first three assumptions.

**FIGURE 3:**
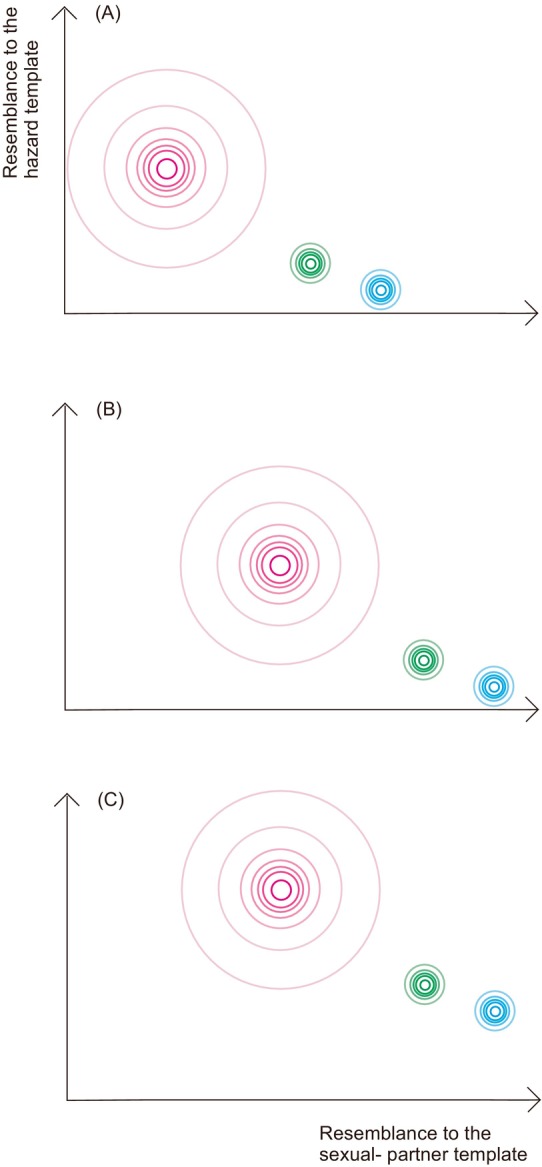
Schematic representation of the Partner‐Hazard Space (PHS). As in a topographic map, each contour line represents the density of objects in the space, with the highest density at the center of each cluster. Objects in blue, green, and red are conspecific opposite‐sex, conspecific same‐sex, and other species (in the conventional perspective), respectively. The perceived position of objects in the space changes with their movement: Static objects (A), passing objects (B), and looming objects (C).

In short, butterflies possess two kinds of templates for other animals: sexual partners that trigger mating behavior, and hazards that trigger escape responses (Figure 3). Past research has shown that moving objects are more attractive than static ones (e.g., Tinbergen 1972; Li et al. 2017) due to their noticeability (Figure 3A,B). Looming objects are perceived as more hazardous (Figure 3). Although additional templates may exist in specific cases, for example, tending partners for myrmecophilous species (Pierce et al. 2002), these are omitted here.

PHS is intentionally conservative: it assumes only cognitive abilities for which empirical evidence exists and excludes additional assumptions about sexual‐rival recognition and species recognition that are commonly invoked in conventional perspectives (Table 3). For this reason, PHS functions as a null hypothesis. From the PHS perspective, experimental results previously interpreted as evidence for species recognition, sexual recognition, and mate choice can instead be understood as the recognition of sexual partners varying in attractiveness (Table 3). It should be noted that PHS does not deny that butterflies recognize sexual rivals and species but claims that such cognitive assumptions are unnecessary at present.

**TABLE 3:** Characteristics of the Partner‐Hazard Space (PHS) and conventional perspective accounts for butterfly behaviors.

	Assumed templates	Assumption of variation in resemblance to each template	Explanatory scope
Partner‐Hazard Space	Sexual partner Hazard	Sexual partner: necessary (variation in attractiveness gives rise to mate choice, including phenomena interpreted as sexual recognition and species recognition in the conventional perspective) Hazard: necessary (larger or looming objects are perceived as more dangerous)	It provides a parsimonious explanation of known butterfly behaviors
Conventional perspective	Sexual partner Sexual rival Species Hazard	Sexual partner: necessary (variation in attractiveness gives rise to mate choice within the conspecific opposite sex) Sexual rival: possibly unnecessary (there may be no social hierarchy within the same sex) Species: necessary (favoring conspecifics is a prerequisite for mate choice and rival recognition) Hazard: necessary (larger or looming objects are perceived as more dangerous)	It requires additional assumptions to explain territorial behavior and other male–male interactions

In some cases, the phenotype of actual conspecific opposite‐sex individuals may not most closely resemble the sexual‐partner template. For receivers, the most important cues of a sexual partner can vary depending on context. A typical case is that males are initially attracted to bright male wings more than to female wings (Lundgren 1977) (Table 1). However, cues specific to conspecific females, such as chemical signals, may more effectively elicit mating behavior at close range. In this situation, conspecific males may more closely resemble the sexual‐partner template at long range, whereas the opposite holds true at short range. Such species‐ or population‐level variations require species‐specific studies.

One might regard PHS as a rather classical ethological perspective. Currently, genetic and physiological approaches are powerful tools for revealing *how* animals recognize stimuli, and they have recently been applied to studies of mate preference in butterflies (e.g., Rossi et al. 2024; VanKuren et al. 2025). However, these approaches do not clarify *what* animals recognize. For example, color vision of the swallowtail butterfly *Papilio xuthus* was demonstrated through associated learning between color papers and food (behavioral experiments), although it had been known that the butterfly has several types of spectral receptors (Kinoshita et al. 1999). Thus, because cognitive abilities cannot be directly observed, we constructed PHS by assuming only those cognitive abilities that are necessary to explain observed behavior.

## 5 Butterfly Behavior From the Perspective of PHS

### 5.1 Intraspecific Interactions

Here, we provide an overview of known intraspecific interactions of butterflies through the lens of PHS. We begin with so‐called “territorial contests” of butterflies, where two males chase each other without physically attacking until one of them flees (Takeuchi et al. 2016). From a conventional perspective, this behavior contradicts the logic of individual selection for the following reason: the male that initiates the chase wastes its own energy or time, while the chased male has no reason either to flee or to respond because neither male can impose costs on the other. Under such circumstances, the optimal response of the chased male would be to ignore the opponent and stay in the territory, and consequently, the optimal strategy of the chasing male would also be to refrain from initiating pursuit. Under this reasoning, territorial contests should not occur at all.

Now consider the same interaction from the PHS perspective. For a territorial male, conspecific males (in the conventional perspective) flying in front of him are objects that resemble the sexual‐partner template more than other animals. At the same time, they also resemble the hazard template to some extent (Figure 3B). However, unless they are looming toward him, the resemblance to the hazard template is generally small enough to be negligible. Therefore, he generally initiates mating behavior toward them.

For an intruder chased by a territorial male, the opponent not only resembles the sexual‐partner template but also resembles the hazard template, as it is actively looming toward him (Figure 3C). The intruder may either flee or also begin chasing the territorial male, depending on the probability that the opponent is a sexual partner (Figure 3C). As this male–male interaction continues, the probability that the opponent is a receptive female decreases either because receptive females tend to copulate soon or because the males accumulate information about their opponent. Eventually, one of the two males gives up chasing. Then, he flees, as there remains a possibility that the opponent still pursuing him is a predator.

This PHS‐based interpretation of territorial contests is supported by a study on the Old World swallowtail, *Papilio machaon* —a species in which PHS might initially seem least applicable, as “territorial” males and intruders physically interact by entangling their legs in mid‐air (Takeuchi 2019). However, Takeuchi et al. (2019) showed that males perform an initial phase of mating behavior (contact the opponent's wing base with their legs) toward both flapping male and female specimens. That is, aerial male–male interactions in this species involve both males performing mating behavior toward each other.

PHS suggests that the term territory may not be appropriate for butterflies, as it implies agonistic contests. The male tactic of waiting for females at specific sites is better described by more neutral terms, such as “perching” (Rutowski 1991). PHS is more realistic than ECH because ECH assumes that males chase all flying objects equally (Takeuchi et al. 2016).

It should be noted that PHS does not deny that males who persistently chase their opponents and occupy perching sites may be favored by sexual selection. If so, traits such as boldness or fearlessness may be subject to selection (Kemp and Wiklund 2004; Kaiser et al. 2019).

PHS also explains other butterfly mating systems. In some species, males do not perch at mating stations but instead fly widely to find receptive females (Rutowski 1991), a strategy known as patrolling. Even in these species, males sometimes chase each other, despite not being territorial (reviewed by Takeuchi et al. 2016). Since no contested resource is involved, this behavior cannot be classified as a contest. However, under PHS, this phenomenon is easily understood: males chase something resembling the sexual‐partner template.

Among patrolling species, some exhibit a unique behavior where males aggregate around a conspecific pupa close to emergence and perform scramble mate competition when a female emerges (reviewed by Takeuchi 2017). A famous example is *Heliconius* species. Deinert (2003) reported that mate competition of *H. hewitsoni* occurs via three stages: (1) competition for a position on a pupa, (2) insertion of abdominal segments under the pupal casing, (3) clasping the female as she emerges. Males with larger wing‐to‐body ratios were suggested to have advantages in this mate competition (Deinert et al. 1994). Since all males cannot sit on the pupa and only one male can mate with the emerging female, competition inevitably occurs even if males do not recognize sexual rivals.

This type of mate competition is in stark contrast to territorial competition. In territorial mating systems, one male monopolizes a mating station (a future mating opportunity) through aerial contests, whereas in pupal mating systems, multiple males do not determine the owner of a pupa (a future mating opportunity). The occurrence of scramble competition over an emerging female indicates that the owner of a pupa is not determined. From the conventional perspective, it seems puzzling that males gathering around conspecific pupae do not expel other males from their vicinity, while males in mating territories do. If males can expel their rivals by aerial interactions, one would expect competition via aerial contests in both systems. On the other hand, PHS provides a plausible explanation. Males regard conspecific males as less attractive partners than conspecific pupae. Therefore, males are less likely to chase each other in this context.

The application of PHS to specific studies is listed in Box 1.

BOX 1 **Applicability of the partner‐hazard space (PHS) to specific studies.**

1. Lederhouse and Scriber (1996) found that males of the black swallowtail *Papilio polyxenes* with innate coloration were more successful in territorial contests than males manipulated to have female‐like coloration. Aerial interactions involving at least one manipulated male lasted longer than those between two males with innate coloration. This result is easily explained by PHS. Manipulated males were perceived as more attractive than innate males, leading the opponent to chase them for longer.
2. Kaiser et al. (2019) reported that males of the speckled wood butterfly *Pararge aegeria* with an artificial red point were more likely to win contests than males with an artificial green point (used for individual identification). PHS provides a possible explanation. Males with a red point are perceived as less attractive, and the opponent chased them more briefly. Consequently, males with a red point tended to “win” territorial contests.
3. Imafuku and Hirose (2016) tested the effects of male brilliant wing coloration of a lycaenid butterfly, *Favonius taxila*, on its territorial behaviors. The authors placed either an unmanipulated (brilliant) or a scale‐removed (non‐brilliant) male specimen on a leaf of a shoot, where territorial males often perched. Territorial males flew near the brilliant specimen more frequently but landed near the non‐brilliant specimen more frequently. The authors concluded the following. (1) Territorial males recognize conspecific males by wing color and are sensitive to them. (2) The brilliant coloration functions as a threat or weapon to rival males and suppresses their establishment of territories. In interpreting these results, the authors assumed that territorial males regard the specimens as conspecific males; however, this is not the simplest interpretation. PHS provides a simpler interpretation. Territorial males may have treated the specimens as conspicuous obstacles. The brilliant (i.e., conspicuous) obstacle was noticed and avoided more frequently than a non‐brilliant one, leading to the observed differences in behavior.
4. Suzuki and Matsumoto (1990) reported a rare mating behavior of the Chinese windmill *Atrophaneura alcinous*. Males fly around larval food plants to locate receptive females. When a male encounters a conspecific mating pair, he clings to the pair and copulates with the female after the previous copulation ends. The original mating male does not prevent the newcomer from mating with the female. This reaction is consistent with PHS: males do not recognize sexual rivals and therefore do not prevent remating.
5. Pinto and Peixoto (2019) stated that contest behaviors in some animals are confusingly similar to mating behaviors and proposed certain characteristics to distinguish between them. They argued that residency effects, that is, the tendency for resident individuals to keep the resource over intruders, serve as evidence of contest behavior. However, this phenomenon can occur in same‐sex sexual behaviors. For example, residents at a mating station have experienced interactions without being attacked by their opponent there, whereas intruders lack such information. Consequently, residents may perceive lower predation risk at the site, leading them to chase intruders longer and thereby increasing their “contest success” (residency effects). Furthermore, PHS points out that the dichotomy of contest and mating behaviors is not always appropriate. Such a dichotomy assumes that animals can recognize sexual partners and rivals. In some animals, however, such rival recognition may not exist.

### 5.2 Interspecific Interactions

Here we provide an overview of reported interspecific interactions of butterflies through the lens of PHS.

Territorial male butterflies are known to chase not only conspecifics but also a variety of passing objects, including other butterfly species, dragonflies, wasps, birds, and even falling leaves or thrown pebbles (Tinbergen 1972; Alcock and O'Neill 1986; Lederhouse et al. 1992; Ravenscroft 1994; Dreisig 1995; Fischer and Fiedler 2001; Ide 2004; Pinzari and Sbordoni 2013; Takeuchi 2019; Biswas et al. 2023). Generally, the duration of chases involving heterospecifics is much shorter than those involving conspecifics. This difference has sometimes been regarded as evidence of species recognition. However, within the PHS framework, these results indicate that butterflies chase something resembling the sexual‐partner template to varying degrees. Consistent with this interpretation, Biswas et al. (2023) reported that territorial males of the Indian crow butterfly *Euploea core* chase conspecific males for longer than other butterfly species and that they chase other butterflies with dark coloration (similar to conspecifics) for longer than those with bright coloration.

Several studies have reported reproductive interference between related species (Obara and Majerus 2009; Friberg et al. 2013; Ohsaki et al. 2020; Ohata 2025). In *Pieris* species, males of the green‐veined white *P. napi* chase the females of *P. melete* more frequently than conspecific females because *P. melete* females are larger than conspecific females (Ohata 2025). This may cause habitat segregation between the two species. There are reports of hybridization between congeneric species (Taylor 1972; Dupuis and Sperling 2016; Ryan et al. 2018) and, more rarely, a copulation between species belonging to different families (Yoshioka and Amino 2025). PHS can explain these phenomena. Related species are generally closer to the sexual‐partner template than more distantly related species and, in certain situations, may even be closer than conspecifics. This increases the likelihood of interspecific sexual interactions.

## 6 Discussion

Although PHS is grounded in proximate mechanisms, it offers a valuable assumption for sexual selection theories. In Fisher's (1930) runaway model, female mating preferences are not subject to direct selection but evolve purely because they are genetically correlated with the favored male trait. However, theoretical studies have shown that such runaway coevolution easily declines if mate choice incurs even small fitness costs, such as reduced fecundity or increased predation risks (Pomiankowski 1987; Kokko et al. 2002). These models assume that females choose among conspecific males, and the only benefit of preferring exaggerated traits is that their sons will inherit those traits and thus be more attractive. However, if animals must identify mates from a broader pool of animals including conspecifics of both sexes and heterospecifics, sexual ornaments transmit vital information: “I am a sexual partner.” Under such circumstances, preference for these traits confers the direct benefit of securing a mating with the conspecific opposite sex. Indeed, same‐sex sexual behavior and reproductive interference are observed in various taxa (Gröning and Hochkirch 2008; Bailey and Zuk 2009; Scharf and Martin 2013; Shuker and Burdfield‐Steel 2017), which is consistent with imperfect mate recognition.

From the perspective of PHS, butterflies have to find their potential mates among a variety of objects that resemble sexual partners. Since the sensory mechanisms used to detect the conspecific opposite sex are imperfect, any traits advertising sexual availability to the conspecific opposite sex are favored by sexual selection because they increase the noticeability of potential mates (Prum 2010). Likewise, preference for such traits by the potential mate is also favored, as it reduces detection costs and fruitless sexual interest toward the same sex and heterospecifics, including potential predators. In this context, Figures 1 and 2 indicate that salient sexual traits (including dissimilarity from sympatric related species) of butterflies function as signals conveying “I am a sexual partner” to the conspecific opposite sex. Consequently, the evolution of conspicuous butterfly coloration does not necessarily require a correlation between ornament expression and genetic quality.

We propose some research directions. As shown in Table 2, there is a limited number of experiments well suited for investigating sexual recognition, where complete specimens of males and females are presented and the phases of mating behavior exhibited by subject butterflies were recorded. This may reflect a tendency among researchers to take sexual recognition abilities for granted. More experiments testing sexual recognition abilities using various species are required to confirm the generality of PHS.

The assumptions of PHS can be visualized experimentally in the following way. From the perspective of PHS, male–male aerial interactions (often interpreted as contests) are an initial phase of mating behavior. Since male copulation attempts (bending their abdomen to the partner) occur after the partner alights, these male–male interactions rarely proceed to a copulation attempt. Perhaps this is why male–male aerial interactions have not been regarded as same‐sex sexual behavior. Therefore, when a stationary specimen of the conspecific male is presented to males engaging in the aerial interaction, PHS predicts that they are more likely to show advanced mating behavior (such as copulation attempt) toward it than males not engaging in the interaction. This prediction is based on the assumption that the former males have already been sexually activated.

As explained in our formulation of PHS, it functions as a null hypothesis, positing that butterflies do not recognize sexual rivals and species (Table 3). PHS does not deny that butterflies have these abilities but claims that the assumption of such abilities is unnecessary at present. If there are butterfly lineages that appear to recognize sexual rivals or species, researchers can formulate alternative hypotheses to challenge PHS. PHS is falsifiable because it would be refuted if researchers find behaviors that cannot be explained without assuming sexual or species recognition abilities. It will be exciting to investigate which lineages have evolved such capabilities and to what extent butterflies possess them. We point out two candidate examples.

Bennett et al. (2012) reported possible mate guarding behavior in the checkerspot butterfly *Euphydryas editha* . In this study, a male that copulated with a female remained positioned behind the female for 7–14 min after copulation. The authors mentioned that this position appeared to prevent other males from approaching the female. However, the results could also be explained as the male having merely rested. If males positioned behind a female actively prevent her re‐mating (for instance, by re‐inserting their genitalia into the female's copulatory opening) when other males approach, this would provide evidence for rival recognition.

Males of *P. napi* transferred more ejaculate to a female under conditions of high male density than under low male density (Larsdotter Mellström and Wiklund 2009, 2015). Although an increase in ejaculate size does not necessarily indicate recognition of sexual rivals because ejaculate size may vary with other factors such as flight activity (Vande Velde et al. 2012), it is possible that males of *P. napi* do recognize sexual rivals. This species is worth studying further.

If rival recognition has evolved in some butterfly lineages, an important question is why it appears to be absent in many others. Butterflies generally lack specialized structures for physically excluding rivals, such as teeth or horns, which may reduce the benefits of the evolution of rival recognition compared with taxa that engage in direct contests.

## 7 Conclusions

As we have discussed, researchers have often misinterpreted quantitative differences in behavior (intensity or frequency of courtship) as evidence of qualitative cognitive abilities, such as sexual recognition or species recognition. In the present study, we integrate inter‐sexual, intra‐sexual, and interspecific interactions within the context of mate preference. By doing so, we provide a general explanation of butterfly behavior.

In this paper, we limit the applications of PHS to butterflies because forms of intra‐ and interspecific interactions vary across animal taxa. However, the general approach underlying PHS is unlikely to be limited to butterflies. We expect that researchers studying other taxa, for example, moths and other insects, will reexamine studies on sexual and species recognition based on simpler cognitive assumptions.

## Author Contributions

**Tsuyoshi Takeuchi:** conceptualization (lead), funding acquisition (lead), investigation (lead), methodology (lead), project administration (lead), validation (lead), visualization (lead), writing – original draft (lead), writing – review and editing (equal). **Daisuke Muramatsu:** visualization (supporting), writing – review and editing (equal).

## Funding

This work was supported by Grants‐in‐Aid for Scientific Research (JP19K06859 and JP25K09774) from the Japan Society for the Promotion of Science.

## Conflicts of Interest

The authors declare no conflicts of interest.

## Data Availability

The authors have nothing to report.
